# Supplementary material for: Graphene-like nanoribbons periodically embedded with four- and eight-membered rings
Source: Nat Commun. 2017 Mar 31;8:14924. doi: 10.1038/ncomms14924 (PMC5381008; doi:10.1038/ncomms14924)
Supplement: Supplementary Information — Supplementary Figures, Supplementary Notes and Supplementary References [file ncomms14924-s1.pdf]

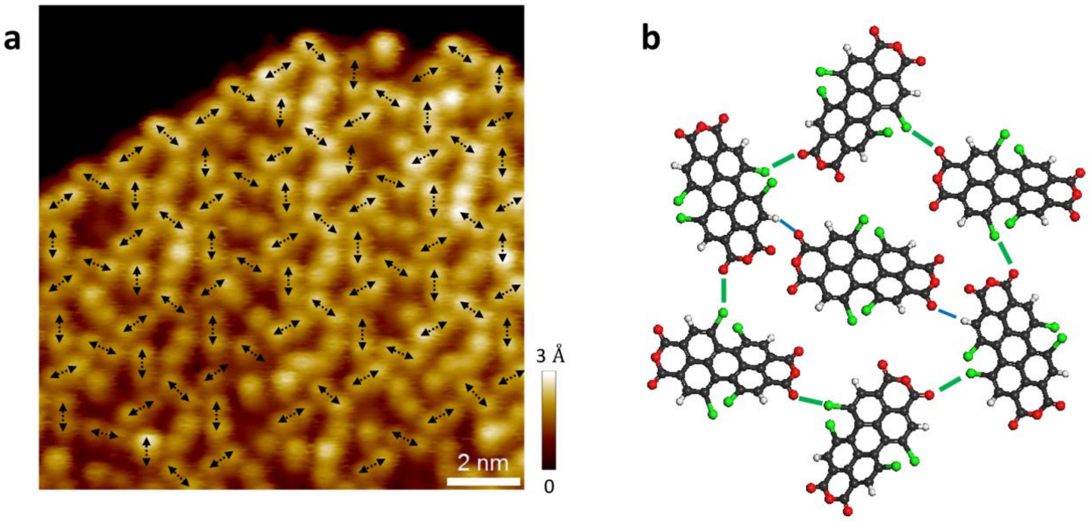

Supplementary Figure 1 | Self-assembled Br_4-PTCDA molecules on Au(111) deposited at room temperature. a, STM image of the Br_4-PTCDA molecules (V = –1.6 V, I = 0.3 nA). Br_4-PTCDA molecules were deposited onto Au(111) surface at room temperature. A double–headed arrow denotes the position of bromine atoms in each molecule. **b**, Optimized structural model of the self-assembled superstructure of Br_4-PTCDA molecules. The average Br···O distance is 3.32 Å, equivalent to the sum of respective van der Waals radii (3.37 Å for Br···O).

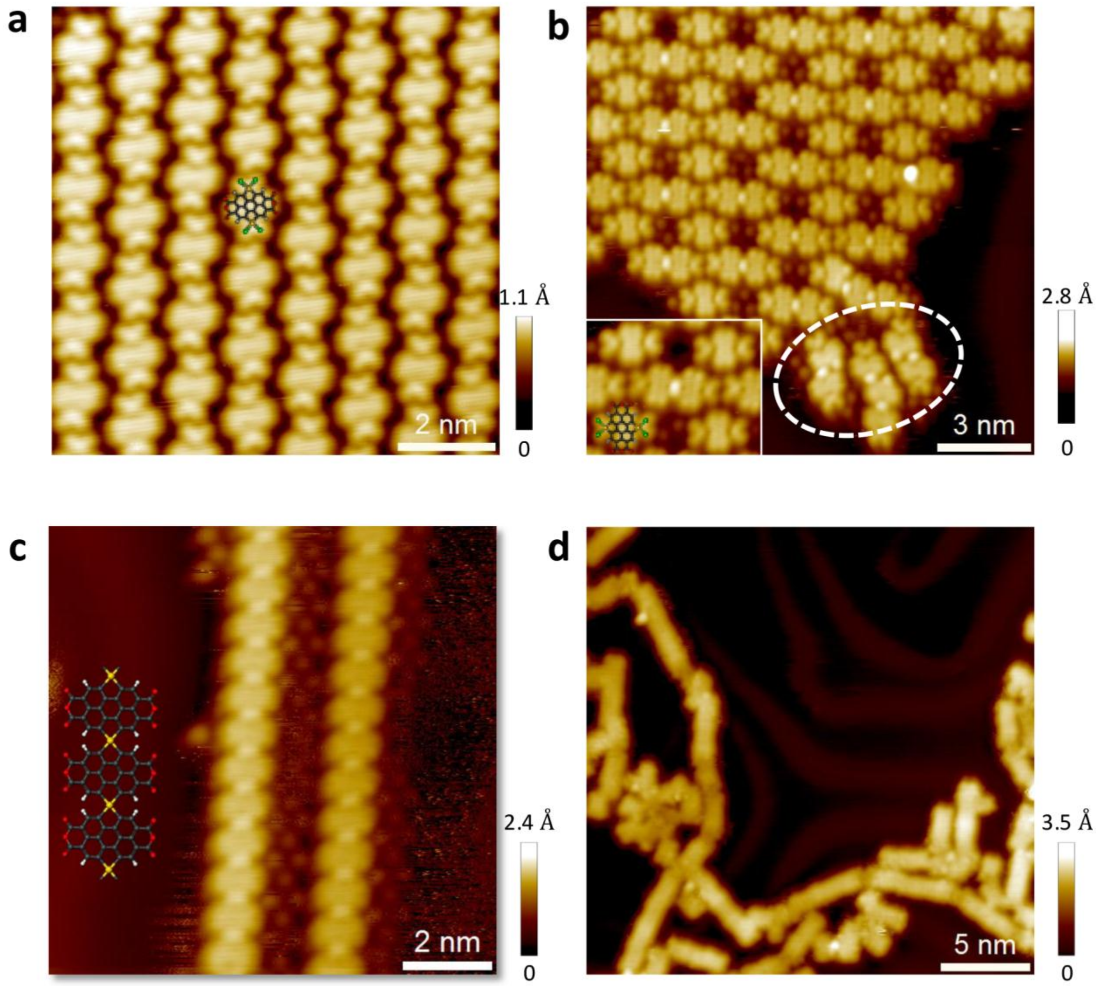

Supplementary Figure 2 | Synthesis of graphene-like nanoribbons from Br_4-PTCDA molecules. a, STM image of the self-assembled structures of PTCDA-Au_2-Br_4 hybrids on Au (111) at 100 °C (V = −0.3 V, I = 1.8 nA). **b,** Stable dimers formed after the formation of Au-C bonds at 160 °C (V = −1 V, I = 0.2 nA). **c,** STM image of linear polymeric chains linked by gold atoms (V = −1 V, I = 0.6 nA). **d,** Graphene-like nanoribbons after the sample was heated to 360 °C (V = −1.2 V, I = 0.5 nA).

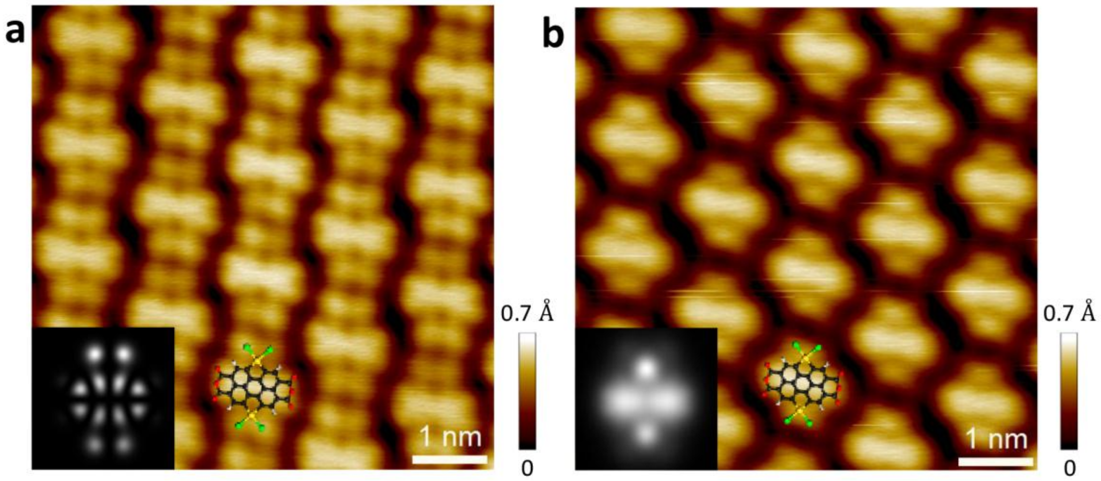

Supplementary Figure 3 | STM images of the PTCDA-Au_2-Br_4 hybrids at negative and positive bias voltages. a, STM image of PTCDA-Au_2-Br_4 hybrids on Au (111) at 100 °C (V = −1.6 V, I = 0.06 nA). The inset shows the DFT-based simulation of the PTCDA-Au_2-Br_4 hybrid at negative bias voltage. **b,** STM image of PTCDA-Au_2-Br_4 hybrids taken with the same tip (V = 1 V, I = 0.06 nA). The inset shows the DFT-based simulation of the PTCDA-Au_2-Br_4 hybrid at positive bias voltage.

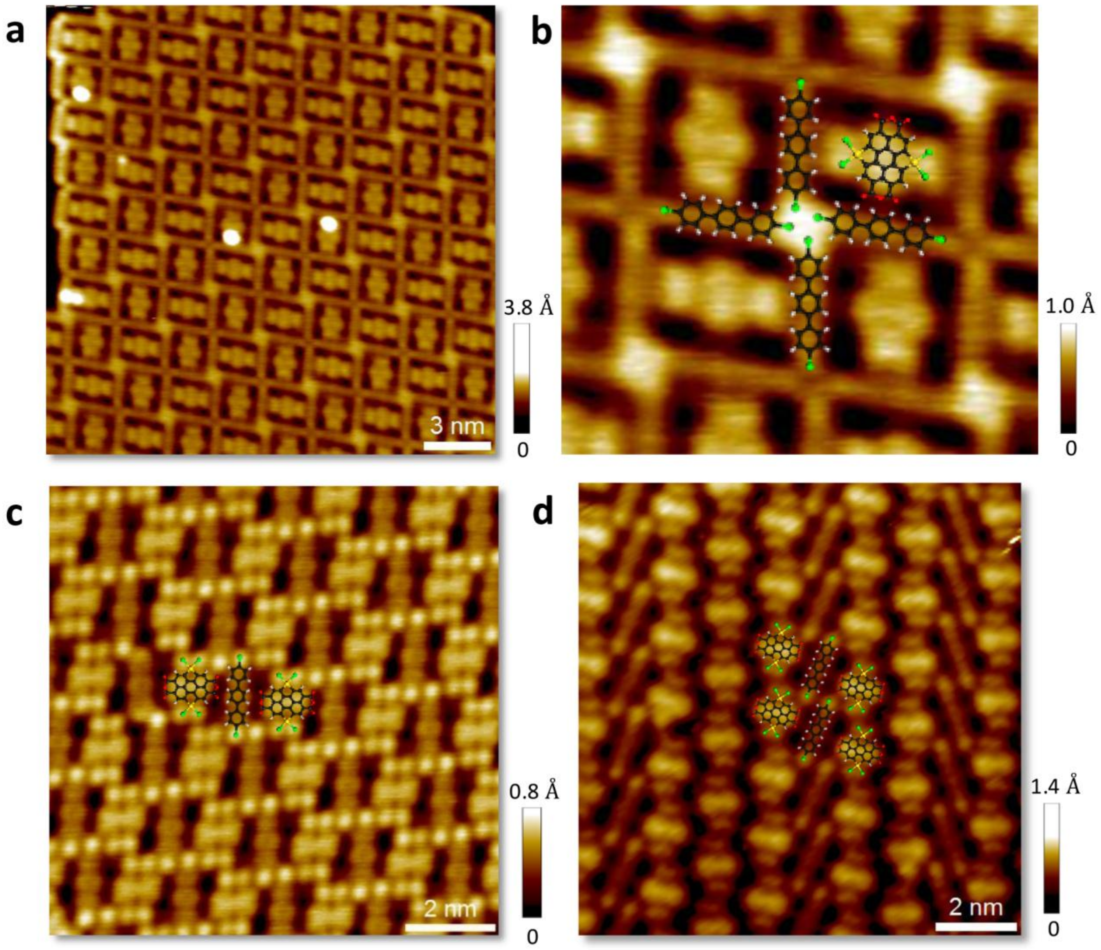

Supplementary Figure 4 | Different types of self-assembled superstructures at different molecular coverages. a, STM image of a well-ordered network formed by PTCDA-Au_2-Br_4 hybrids and DBTP molecules at about 1:2 ratio (V = –2 V, I = 0.1 nA). **b**, STM image showing a quartet node of windmill shape formed by four DBTP molecules. **c**, STM image obtained at about 1:1 growth ratio of Br_4-PTCDA molecules : DBTP molecules (V = –1.6 V, I = 0.3 nA). **d**, STM image of PTCDA-Au_2-Br_4 hybrids and DBTP molecules at about 2:1 ratio (V = –2 V, I = 0.1 nA).

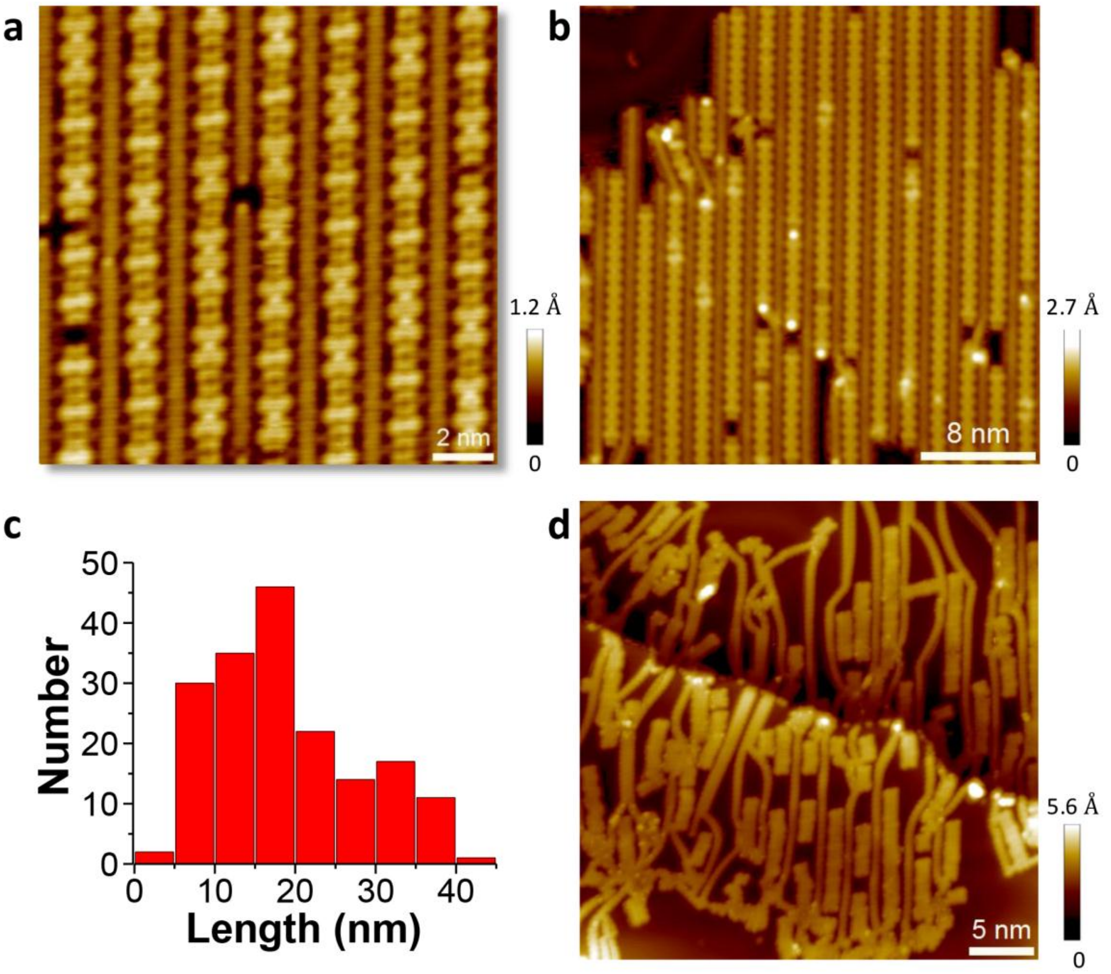

Supplementary Figure 5 | Synthesis of graphene-like nanoribbons with assistance of the PPP polymers. a, STM image of PPP polymers formed after dehalogenation at 160 °C with PTCDA-Au oligomers lying in between (V = –1.2 V, I = 0.3 nA). **b**, STM image of 280 °C-annealed sample (V = –1.8 V, I = 0.1 nA). **c**, The length distribution of the PTCDA-Au polymeric chains based on the analysis of a total of 178 ribbons. **d**, STM image of graphene-like nanoribbons comprising four- and eight-membered rings formed after C-Au bond cleavage and cyclodehydrogenation at 360 °C (V = –2 V, I = 0.1 nA).

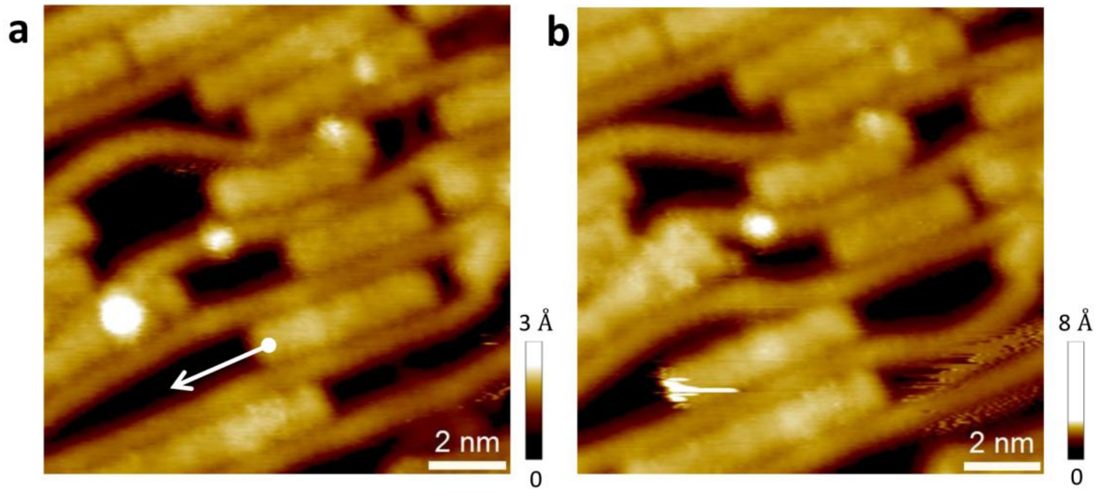

Supplementary Figure 6 | Manipulation of the graphene-like nanoribbons by STM tip. **a, b,** STM images of the same region before (**a**) and after (**b**) STM tip manipulation (V = −1.2 V, I = 1.1 nA). White arrow denotes the trace of the tip.

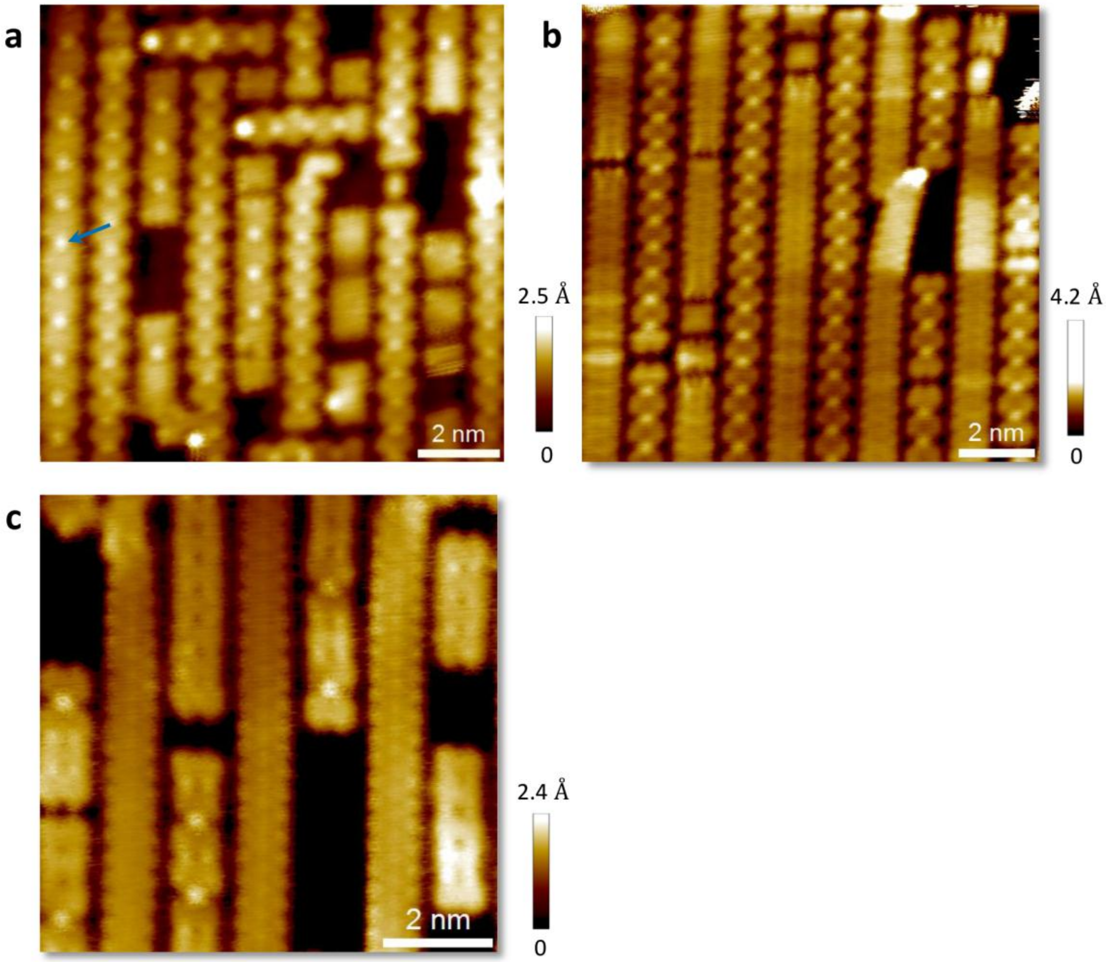

Supplementary Figure 7 | Templated on-surface synthesis of graphene-like nanoribbons using armchair graphene nanoribbons. a, STM image of PTCDA-Au polymers and Au-DBBA hybrids at 200 °C (V = –2 V, I = 0.1 nA). **b**, 7-GNRs formed after cyclodehydrogenation (V = –1 V, I = 0.2 nA). **c,** Graphene-like nanoribbons comprising four- and eight-membered rings formed after the Au-C bond cleavage and cyclodehydrogenation at 380 °C (V = –0.4 V, I = 1.5 nA).

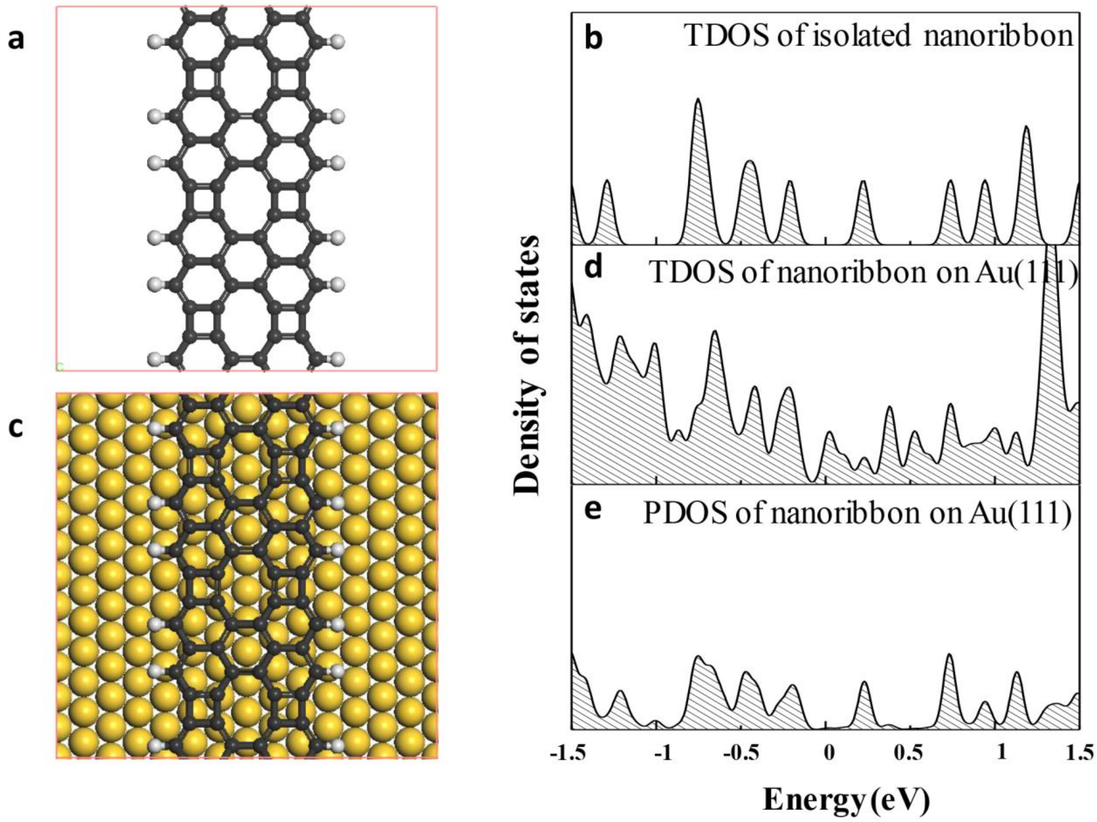

Supplementary Figure 8 | Density of states (DOS) of the isolated nanoribbon and nanoribbon adsorbed on Au(111) from DFT calculations. a, c, Optimized structural models of the isolated nanoribbon and nanoribbon adsorbed on Au(111) surface. For simplification of the calculations, the anhydride groups are not involved. **b**, Total density of states (TDOS) of the isolated nanoribbon. **d**, Total density of states of the nanoribbon adsorbed on Au(111) surface. **e,** Density of states projected on the nanoribbon.

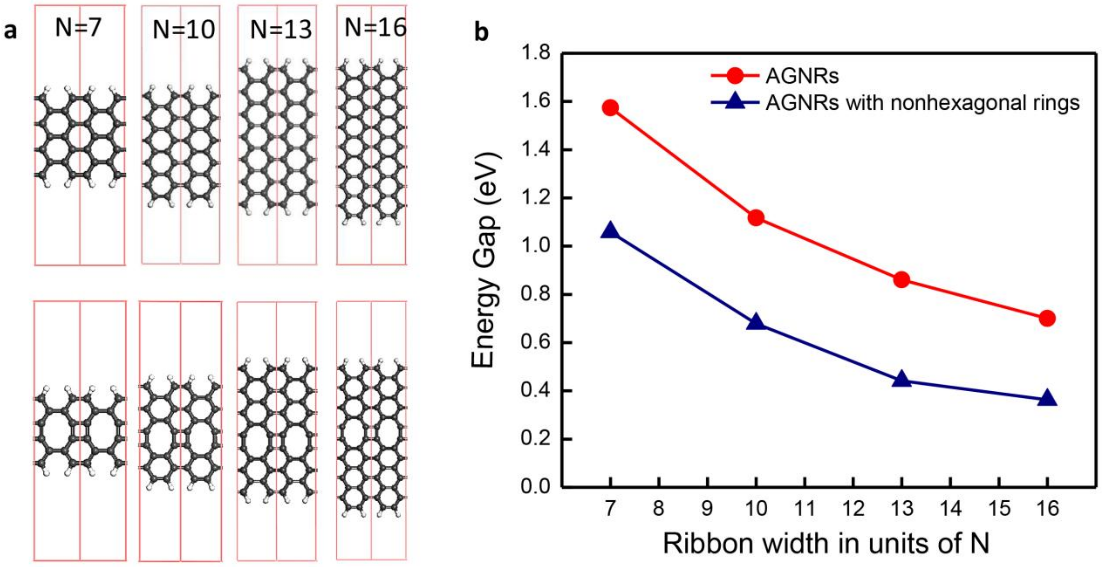

Supplementary Figure 9 | Variation of band gaps with the width of AGNRs. a, Optimized structures of the N=3p+1 family of AGNRs (N is the number of dimer lines across the ribbon, and p is an integer) and corresponding nanoribbons embedded with four- and eight-membered rings. **b,** Calculated band gaps of AGNRs (red) with and without nonhexagonal rings (blue) decreasing as a function of ribbon width in units of N, respectively.

Supplementary Note 1: Self-assembled Br_4-PTCDA molecules deposited at room temperature

The halogen bond, a non-covalent interaction attributed to the polarized chlorine, bromine, or iodine molecular substituents, has been exploited to tailor the self-assembly of small molecules[1-2]. In our experiment, the Br_4-PTCDA molecules were deposited onto Au (111) surface held at room temperature under ultrahigh-vacuum (UHV) conditions. For a single molecule, only two bright spots were observed in the STM image, which correspond to the bromine atoms. As illustrated in Supplementary Fig. 1a, a hexagonal halogen-bonded superstructure was formed by electrostatic interaction between the polarized bromine substituents and the lone pair electrons of oxygen atoms of neighboring carbonyl groups. In the porous area enclosed by six intact molecules, another Br_4-PTCDA molecule interacts with surrounding molecules by hydrogen bonds, forming the different configurations in different pores. We reproduced the molecular model for the observed structure by DFT calculations with van der Waals correction considered (Supplementary Fig. 1b).

Supplementary Note 2: Synthesis of graphene-like nanoribbons from Br_4-PTCDA molecules

By annealing at 100 °C for 10 min, the Br_4-PTCDA molecules converted to PTCDA-Au_2-Br_4 hybrids. The gold atom linked with two bromine atoms can be distinguished as a bright protrusion in the PTCDA-Au_2-Br_4 hybrids. A well-ordered self-assembled structure is formed by hydrogen and halogen bond interaction between the gold-organic hybrids (Supplementary Fig. 2a). The Au-Br bonds are partially cleaved at 160 °C, producing the stable dimers linked by gold atoms (Supplementary Fig. 2b). The dehalogenated intermediate with one gold atom at bay area was unexpectedly observed (circled in Supplementary Fig. 2b). After complete dehalogenation at 220 °C, the PTCDA radicals colligate into linear polymer chains connected by gold atoms at bay regions. In Supplementary Fig. 2c, the bright protrusions identified as gold atoms appear in the polymer chains with a periodicity of 8.1 ± 0.1 Å in excellent agreement with the DFT calculation (8.18 Å). By further heating to 360 °C for 10 min, the C-Au bonds are cleaved and gold atoms are released from the PTCDA-Au polymers. The simultaneous cyclodehydrogenation finally results in the formation of four- and eight-membered rings between two adjacent perylene backbones (Supplementary Fig. 2d). But only some fragments of graphene-like nanoribbon are obtained with most of radicals linked together intricately. The experimental results indicate that the instability of anhydride groups at high temperature could obstruct the formation of graphene-like nanoribbons in good quality.

Supplementary Note 3: Bicomponent self-assembled superstructures modulated by different molecular coverage

At different molecular coverage, different types of superstructures can be observed (Supplementary Fig. 4). Since covalently-bonded halogen atoms have both positive and negative electrostatic parts, four DBTP molecules can form the quartet nodes of windmill shapes by means of Br⋯Br bonds and Br⋯H bonds[3]. In Supplementary Fig. 4a, the porous area formed by four DBTP molecules is large enough to encage one PTCDA-Au_2-Br_4 hybrid in a chiral direction. The existence of a PTCDA-Au_2-Br_4 hybrid makes each DBTP molecule participate in the quartet nodes only at one end with the other end unconstrained. In Supplementary Fig. 4c, the primary noncovalent interaction exists between the terminal bromine atoms of DBTP and the lone pair electrons of oxygen atoms of neighboring carbonyl groups. These different structural features regulated by different growth ratios of two molecules are a result of competition between Br⋯Br bonds and Br⋯H bonds.

Supplementary Note 4: Synthesis of graphene-like nanoribbons with assistance of the armchair graphene nanoribbons

We used 10,10′-dibromo-9,9′-bianthryl (DBBA) molecules to produce graphene nanoribbons to provide the 1D constraint on the PTCDA intermediates. Organometallic intermediate always plays an important role in the Ullmann coupling reactions. Supplementary Figure 7a reveals the existence of Au-DBBA intermediates during the on-surface synthesis of GNRs. The hydrogen bond interaction between two adjacent polymer chains contributes to the stabilization of the Au-DBBA hybrids. On annealing at 280 °C, straight GNRs were obtained after cyclodehydrogenation still with some nanoribbon fragments not linked together. Since the temperature is not high enough, the PTCDA radicals are still linked by gold atoms[4] (Supplementary Figure 7b). Graphene-like nanoribbons comprising four- and eight-membered rings can be formed after further heating to 380 °C for 10 min (Supplementary Figure 7c). The dark spots along the central axis of graphene-like nanoribbons have the same repetition period as shownin Fig. 2e.

Supplementary Note 5: The effect of four- and eight-membered rings on band gaps of AGNRs

The variation of band gaps of armchair graphene nanoribbons (AGNRs) exhibits three family behaviors. We chose the 3p+1 (p is an integer) family of AGNRs to study the effect of nonhexagonal rings on AGNRs. Four- and eight-membered rings are periodically embedded into the AGNRs. With the same calculation parameters, a distinct band gap narrowing can be observed regardless of the variation of width (Supplementary Figure 9b).

Supplementary References

1. Auffinger, P., Hays, F. A., Westhof, E. & Ho, P. S. Halogen bonds in biological molecules. *Proc. Natl Acad. Sci.* USA **101**, 16789–16794 (2004).
2. Voth, A. R., Khuu, P., Oishi, K. & Ho, P. S. Halogen bonds as orthogonal molecular interactions to hydrogen bonds. *Nat. Chem.* **1**, 74-79 (2009).
3. Chung, K-H. *et al.* Polymorphic porous supramolecular networks mediated by halogen bonds on Ag(111). *Chem. Commun.* **47**, 11492–11494 (2011).
4. Blankenburg, S. *et al.* Intraribbon heterojunction formation in ultranarrow graphene nanoribbons. *ACS Nano* **6**, 2020–2025 (2012).
